# Camouflage through colour change: mechanisms, adaptive value and ecological significance

**DOI:** 10.1098/rstb.2016.0342

**Published:** 2017-05-22

**Authors:** Rafael C. Duarte, Augusto A. V. Flores, Martin Stevens

**Affiliations:** 1 Centro de Biologia Marinha, Universidade de São Paulo, São Sebastião, Brazil; 2 Programa de Pós-Graduação em Biologia Comparada, Faculdade de Filosofia, Ciências e Letras de Ribeirão Preto, Universidade de São Paulo, Ribeirão Preto, Brazil; 3 Centre for Ecology and Conservation, College of Life and Environmental Sciences, University of Exeter, Penryn Campus, Penryn, Cornwall TR10 9FE, UK

**Keywords:** colour change, camouflage, behaviour, chromatophores, predator–prey interactions

## Abstract

Animals from a wide range of taxonomic groups are capable of colour change, of which camouflage is one of the main functions. A considerable amount of past work on this subject has investigated species capable of extremely rapid colour change (in seconds). However, relatively slow colour change (over hours, days, weeks and months), as well as changes arising via developmental plasticity are probably more common than rapid changes, yet less studied. We discuss three key areas of colour change and camouflage. First, we review the mechanisms underpinning colour change and developmental plasticity for camouflage, including cellular processes, visual feedback, hormonal control and dietary factors. Second, we discuss the adaptive value of colour change for camouflage, including the use of different camouflage types. Third, we discuss the evolutionary–ecological implications of colour change for concealment, including what it can tell us about intraspecific colour diversity, morph-specific strategies, and matching to different environments and microhabitats. Throughout, we discuss key unresolved questions and present directions for future work, and highlight how colour change facilitates camouflage among habitats and arises when animals are faced with environmental changes occurring over a range of spatial and temporal scales.

This article is part of the themed issue ‘Animal coloration: production, perception, function and application’.

## 1. Introduction

Many pioneers of evolutionary biology, including Wallace and Poulton, spent considerable time discussing animal coloration and describing the types of camouflage that may exist [1,2], providing key examples of natural selection. Early experts were also aware that individuals of many species could change colour, and Poulton even conducted experiments into the mechanisms and function of this (e.g. [3]). Ever since, colour change has been a valuable system to study both the adaptive value of camouflage and the physiological processes shaping animal form and diversity [4].

Colour change occurs over multiple timescales. Cephalopods like cuttlefish can change rapidly in seconds, many fish change in minutes, some crabs over a period of hours, caterpillars over days and weeks, and certain Arctic animals over months [5,6]. In many species, a fundamental reason for colour change is that it allows individuals to modify their appearance to provide camouflage tuned to the habitat where they live, and to cope with environmental changes occurring over a range of spatial and temporal scales. For example, rapid changes in appearance may enable animals to cope with fine-scale temporal and spatial heterogeneity, whereas slower changes are often associated with more predictable long-term environmental variation [5].

Spanning over 120 years, substantial research work has sought to understand the functions and mechanisms of colour change for camouflage. However, numerous key questions remain, including: What are the mechanisms of colour change across species? What are its physiological costs? What is the adaptive value provided? and How does colour change relate to intraspecific diversity and various ecological processes? Here, we discuss the mechanisms and functions of colour change for camouflage and identify key questions for future work. Our synthesis differs from recent reviews on a similar theme that have either discussed the general ecological factors favouring flexible animal defences (from startle displays to camouflage), but not covered mechanisms [5], or focused on colour change in one specific group (e.g. crabs) [6]. Here, we provide an integrative synthesis of the mechanisms that underpin colour change for camouflage, its adaptive value, and the evolutionary–ecological factors and implications of colour change for concealment.

## 2. Mechanisms and control of colour change

### (a) Cellular basis

**Figure 1.**
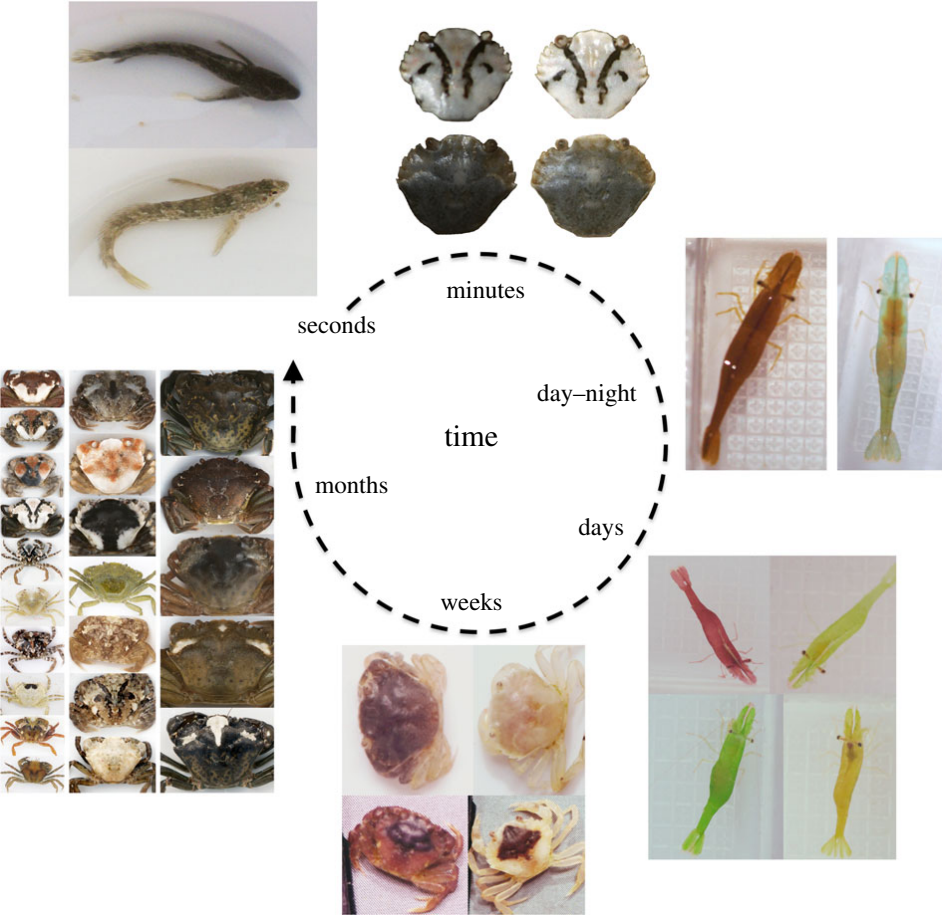
Animals can modify their appearance over varied timescales. Some fish, like rock gobies (*Gobius paganellus*), change brightness and colour in less than a minute. Shore crabs (*Carcinus maenas*) change brightness over 2 h, becoming darker on black (left images) and lighter on white backgrounds (right images). Chameleon prawns (*Hippolyte varians*) undergo day–night changes from their diurnal type (here a brown morph) to blue–grey at night. Colour change also often occurs over longer periods. Red chameleon prawns change to green when on green seaweed for 20 days (top), and begin to change from green to yellow–red when on red seaweed (bottom; though this direction of change seems slower). Shore crabs substantially change appearance as they moult over weeks and months. Here, the top individual changes from dark to light post-moult after being kept on a white substrate. The bottom images show an individual changing colour and pattern with moult after having been kept on a light yellow substrate. Changes also occur with ontogeny, with juvenile crabs (left column) highly diverse in appearance, but variation declining from subadults (middle column) to adults (right column).

The basis of colour change has been studied for a considerable time (e.g. [7]), and the varied mechanisms involved have been reviewed in detail [8–11], primarily with regard to endocrine and cellular control. Colour change can involve a range of mechanisms, and these can be quite different between vertebrates and invertebrates (see [8–11]). However, it has been studied most with regard to changes in the state and abundance of pigment-containing chromatophore cells. These cells can respond directly to light (a primary response) or via visual system pathways (a secondary response), with the latter being more relevant to camouflage. Broadly, secondary responses can involve two processes and occur over a variety of timescales (figure 1). Physiological colour change occurs over seconds, minutes and hours, and involves dispersion and aggregation of pigment within chromatophores (involving pigment granules and pigment-containing vesicles in the chromatophores of invertebrates and vertebrates, respectively) [8,9,11]. This can include rapid changes brought about by neuromuscular action directly on the cells, as in cephalopods [8], or slower endocrine pathways, such as in arthropods, or a combination of neural and endocrine signals in some fish and reptiles [11]. Colour change can also involve morphological processes, including changes in the number and proportion of chromatophore types and pigment content [8,9]. This includes moulting in many species (e.g. crabs and caterpillars [6,12]), and can occur over days, weeks and months, and be dramatic in terms of appearance [6].

A significant body of work, especially in crabs, fish and amphibians, has investigated the nature and control of chromatophores, and described the types of cell that exist. These include melanophores (black/brown melanin pigment), erythrophores and xanthophores (red and yellow, respectively, with pteridine and carotenoid pigments), and leucophores or iridophores (involving purines producing especially white and blue colours mainly through light reflection) [8–10]. Much is known about these cells and their control. However, most past work on camouflage scored individual chromatophore states based on a pigment-dispersion index and the challenge is to relate changes in chromatophore state and abundance to objective changes in the animal's appearance, and especially quantifying actual camouflage match (but see e.g. [13–17]). The role of molecular pathways and genetic control is somewhat less known (but see [9,10]).

Although a camouflage function of colour change is often assumed rather than quantified, numerous experiments demonstrate that many species change using these cells through assessing the pattern, colour and brightness of the background. For example, many amphibians become darker on a black background due to melanosome dispersal and iridophore aggregation, with the opposite situation observed on a light background [9]. Similar outcomes and processes occur in invertebrates [16,18–20]. Overall, the exact colour change responses are determined by the combined interacting effects of changes in the state, proportion, abundance and arrangement of chromatophore types [9].

### (b) Metabolic and physiological costs and constraints

Colour change is often assumed to involve physiological costs and energetic expenditure. However, to what extent this is true, and how much it actually impacts on other processes and animal energy budgets is little known. In cephalopods, controlling large numbers of chromatophore cells rapidly and in synchrony continuously over time probably carries a cost that impacts on the individual's energy budget [4]. Pigments used in morphological colour change may also be important for non-camouflage functions, such as immune response and health, representing further constraints (especially if colour change involves a role of diet) [6]. Therefore, metabolic costs and other constraints associated with changing colour and maintaining chromatophore state may be important. One study has at least demonstrated that when guppy fish (*Poecilia reticulata*) are induced to change colour by altering the background, individuals increase their food consumption levels [21]. The implication is that increased food consumption occurs to offset the energetic costs of changing colour. However, while valuable, this finding comprises indirect evidence. Alternative explanations include, for instance, that surplus feeding is a stress response, or that fish need to obtain the pigments required for colour change from their food (i.e. a constraint but not a cost). Experiments taking metabolic respirometry measurements, while inducing individuals to change by presenting them with different visual backgrounds, are needed to quantify energetic expenditure associated with changing colour.

### (c) Role of visual pathways

Most work on how visual information drives change in appearance for camouflage has been undertaken in cephalopods, especially cuttlefish (reviewed in [22]). Such work has shown that cephalopods change their patterns in response to the size, contrast and presence of visual edges and discrete objects, among other factors. Similar studies have also been undertaken in flatfish (e.g. [23]), but manipulative tests of background appearance and camouflage change responses are more sparse in other taxa. In addition to changes in pattern, animals also change colour and brightness (e.g. [15,17]). This potential should be enhanced by an increased ability to resolve greater colour, or via rules of thumb associated with scene brightness. By contrast, poor visual ability may constrain colour change and matching. Furthermore, responses to background brightness are thought to be mediated not by overall light levels, but by the ratio of incident light from around the animal to the light reflected from the substrate [6]. One study on grasshoppers has shown that colour change to darker or lighter forms occurs when the substrate is comparatively light or dark, but not simply when individuals are put into dark containers or bright light [24].

In cephalopods and some fish and reptiles, chromatophore state is under direct nerve control from the visual system, whereas in many other animals it is guided by hormones. A wide range of work on *Uca* crabs and other crustaceans shows a chromatophore response at least partly based on eyestalk (sinus gland) produced hormones (e.g. [25]). However, in *Cancer* crabs, evidence also suggests a role of the optic nerve controlling other non-eyestalk sources of pigment-dispersing hormones [26]. In many vertebrates, especially amphibians, much is known regarding the hormones involved in colour change and how they act on the pigment cells (see [8,9]). Work on other taxa investigating these pathways and specifically into the role of vision would be valuable in understanding the control of colour change and associated constraints. Finally, there is growing evidence that colour change for camouflage may also be partly guided by light-detecting opsin proteins outside the eye, and this is an important area for future work (see summary in [6]).

### (d) Role of diet

**Figure 2.**
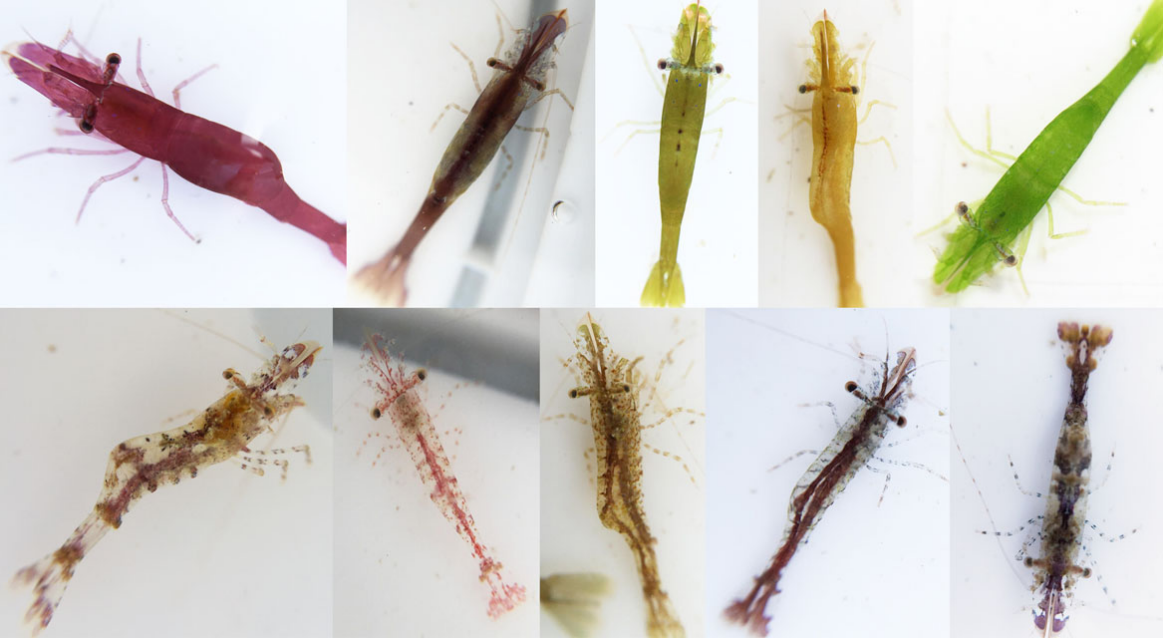
Intraspecific diversity and matching to different backgrounds. The chameleon prawn (*Hippolyte varians*) has considerable intraspecific variation, with multiple relatively homogeneous and interchangeable colour types ranging from red to green (top row), in addition to seemingly fixed transparent morphs (bottom row). These different morphs allow camouflage against different seaweed types.

While visual feedback for colour change is undoubtedly important in many species, a role of diet also exists in some groups, and likely often interacts with vision. For example, diet is known to influence coloration in some spiders [27]. In *Hippolyte* prawns (figures 1 and 2), effective colour change from one morph to another seems to require the presence of real seaweed, rather than colour matched artificial backgrounds [28], although this does not discount a role of vision. In caterpillars, studies (some stemming back to Poulton, e.g. [3]) have shown that, depending on the species, larval coloration is induced by either diet, substrate reflectance or a combination of these [12,29], though the precise mechanisms are uncertain. Further manipulative experiments, changing the visual appearance of the substrate through coloured filters, alongside diet, would enable teasing apart the relative roles of diet and vision.

### (e) Circadian rhythms

Many animals that change appearance also show clear circadian rhythms of colour change. For example, various crabs become lighter at night and darker during the day (perhaps to save energetic costs at night when predation risk is lower) [30], or darker at night and lighter during the day to improve camouflage [20]. These changes do not generally occur simply when individuals are put into the dark, and are in tune with natural day–night cycles, indicating endogenous control modified by long-term changes in light conditions and day length (e.g. [20,25,30]). Other species, such as chameleon prawns (*Hippolyte varians*), also show pronounced changes at night (changing from a green, red or brown diurnal colour to a blue-transparent nocturnal appearance [31]; figure 1). However, these changes seem to involve responses to light conditions over a few hours [32], suggesting a periodic release of hormones [8]. Overall, circadian rhythms may allow animals to achieve camouflage under strikingly different light conditions and times of day, but they may also play a role in other functions, such as protection from ultraviolet light and in thermoregulation.

### (f) Behavioural adaptations

Unlike species known to rapidly change appearance (e.g. certain cephalopods and fish), most animals cannot change immediately but probably over hours, days and weeks [6,11]. This means that if coloration is to effectively provide camouflage in heterogeneous environments, individuals should also behaviourally choose backgrounds that match their appearance. Recent evidence in some species (e.g. birds [33]) shows that individuals can choose backgrounds based on their individual appearance, though the mechanisms by which they do so are unknown. Species that change colour should not have fixed behavioural preferences, however, because they would then often choose inappropriate backgrounds after having changed colour. By contrast, preference should be flexible. For example, a crab newly found on a white background may both turn white and develop preferences for white substrates over time. As yet, we are aware of few tests of such ideas (but see [34,35]). However, evidence from *Hippolyte* prawns and filefish (*Monacanthus chinensis*) shows some evidence of substrate preferences linked to morph types and individual coloration [28,36,37].

## 3. Causes, consequences and ecological implications of colour change for concealment

### (a) The survival benefit of colour change for camouflage

It is widely assumed that predation pressure is the ultimate selective force promoting camouflage. To date, experimental testing of how camouflage strategies mitigate predation comes mostly from studies using model replicas of real animals (e.g. [38]). While these are valuable for testing camouflage effectiveness, the stimuli used are necessarily simplified and do not show the direct survival advantage afforded by camouflage in the real animals. Other studies, such as those of melanism in the peppered moth (*Biston betularia*), have analysed morph frequencies and recapture rates in different habitats (e.g. [39]). Ideally, we need to relate direct quantification of individual-level camouflage to predator vision, and how this equates to predation risk.

Colour-changing species provide an opportunity to assess how changes in individual appearance influence predation risk. Some studies have used mathematical vision models to assess changes in background matching. For example, in cuttlefish, appearance changes may allow efficient concealment of individuals to both di- and trichromatic fish predators [13]. Likewise, colour change in chameleons conceals individuals to the vision of birds and snakes [17]. Adjustments in background matching, assessed using models of predator vision, have also been undertaken in crabs and fish [15,16]. Some studies, e.g. on colour-changing isopods [40], have used tethering experiments to show that survival (recapture) of morphs is higher against matching than non-matching substrates. This approach is valuable because it is closer to testing the actual survival of individuals of differing camouflage in the wild, though future work needs to analyse levels of concealment to predator vision. Overall, direct measurements of how colour change affects the survival of individuals in natural systems represent a major research avenue for the future. Such work should involve predation experiments with real predators and prey, either under field conditions or controlled, but ecologically realistic, laboratory studies. Furthermore, because colour-changing animals exploit a variety of (micro)habitats, the transition from one habitat patch to another, coupled with the time for changes to occur once settled, are critical periods when predation rate is theoretically highest. Therefore, a better understanding of the adaptive value of colour change demands not only testing whether colour matching actually renders lower predation pressure, but also measuring predation rates under transitional non-matching conditions.

### (b) Multiple camouflage strategies

Colour change has a variety of functions, from thermoregulation to sexual signals, and the ability to change should allow animals flexibility in how these are used. Non-camouflage uses of colour change have been well covered elsewhere (e.g. [4,5]), and here we focus on changes in concealment. Camouflage prevents detection or recognition by observers and can be achieved by a number of strategies (see [41]). Various studies have examined how animals employ distinct strategies and their adaptive value, but mostly focusing on species with fixed coloration and using artificial prey. Most past work on colour change considers background matching, whereby the animal resembles the general appearance of the background. By contrast, disruptive coloration breaks up animal outlines and creates false boundaries [41]. Other camouflage strategies include masquerade (resembling objects in the environment to prevent recognition), and transparency. For colour-changing animals, assessment of the camouflage types individuals use and how they provide concealment to different backgrounds are rare, let alone quantifying the presence and effectiveness of strategies such as disruptive coloration. Exceptions include work on cephalopods, which can quickly change between a background matching strategy and one of potential disruptive coloration when individuals are exposed to backgrounds differing in levels of pattern contrast [22]. In *Sepia officinalis*, individuals exhibit ‘uniform’ or ‘mottled’ coloration when placed against low contrast backgrounds, indicating background matching. On the other hand, when placed against strongly contrasting backgrounds, individuals produce a coarse contrasting pattern, composed of spots varying in colour, size and shape, possibly affording a disruptive effect [22]. Many cephalopods are also adept at masquerade. Further work in other species/taxa should consider manipulative experiments placing individuals against substrate types coupled with analysis of their resulting colour patterns. The latter could involve using models of animal spatial vision to calculate levels of disruption to body edges (e.g. [42]) and analysis of body shape and structure to assess similarity to other objects (masquerade). Furthermore, investigations of the colour pattern and body forms adopted by free-ranging individuals would help in understanding under what contexts different camouflage strategies are adopted.

Most animals are exposed to multiple predators, potentially differing in visual ability and strategies for prey detection. Colour change may thus be an important mechanism for individuals to adjust their camouflage when facing different predator threats. Dwarf chameleons, for example, change depending on the predators' visual capabilities, showing greater changes in background colour matching when presented with birds versus snakes. They appear, however, more camouflaged to the snake, as this trichromatic predator has poorer colour discrimination than tetrachromatic birds [17]. Changes in predation risk with ontogeny in some crustaceans may also be an important selective force maintaining high colour polymorphism (see §3c below [43–45]). Further experiments could include testing short-term changes in animals such as cuttlefish, which are known to show divergent responses to different types of predator [46], and also how appearance is altered in animals that change colour over longer periods when exposed to different levels of predator risk.

### (c) Ontogenetic changes and polymorphism

Many animals move from one habitat to another over their life in a predictable way, and this can be coupled with changes in coloration. Ontogenetic changes in colour occur in many taxa and for a variety of reasons, but have perhaps been most widely investigated for camouflage in crustaceans [47]. For example, some colour-changing crabs shift from occupying intertidal habitats as juveniles to subtidal algal patches when larger/adult [34]. In many species, larger crabs are subjectively less cryptically patterned than juveniles [45] (figure 1). Presumably, because these young stages have not yet reached a refuge size against most of their predators, crypsis is prevalent. By contrast, lower plasticity in adults may be a result of moving to more homogeneous background environments, or weaker predator selection for camouflage if individuals are more defended [45]. Finally, physiological constraints may also limit chromatic variation among adults. For larger crustaceans, thicker exoskeletons are less transparent and can impair the potential for colour change.

Beyond ontogenetic shifts, there are many examples of high intraspecific variation and polymorphisms across a wide array of animal groups (figure 2), which may have a number of explanations. These can arise under selection for various functions, but camouflage is one of the most common. In many decapod crustaceans, including crabs and lobsters [44,48], juveniles exhibit higher colour variation than adults, and subjectively achieve camouflage in a wider variety of (micro)habitats (figures 1 and 2). This is paralleled in other groups (e.g. snakes [49]). There are a number of possibilities here regarding why polymorphism in juveniles is high and how it arises. First, to what extent early-stage phenotypic diversity is due to genetic variation versus plasticity is poorly understood—in some groups, the mechanisms may vary from one species to the next [24,50]. Second, why juveniles are so variable is an unresolved question. Greater diversity may reflect habitat heterogeneity being high at small spatial scales—individual phenotypes may match different habitat patches. Juvenile shore crabs, for example, have greater variation in more diverse habitat types [44]. It would be valuable to test whether individuals change their specific patterns to resemble particular background patches, or simply increase general pattern expression when occurring in more variable habitats. Another selective force promoting polymorphism may be that polymorphism interferes with predator search image formation, resulting in apostatic selection. There is some evidence that colour polymorphism may reduce predation rates on juvenile rock crabs (*Cancer irroratus*) inhabiting heterogeneous habitats, whereby predator exclusion led to a higher proportion of the adult-like (drab brown) morphs compared with control conditions [43]. It is unclear, however, to what extent juvenile crabs changed colour in their microhabitats. Experiments similar to those of Bond and Kamil [51], but with real prey phenotypes rather than artificially generated ‘moths’, would be valuable to investigate whether polymorphisms interfere with predator search image formation.

### (d) Morph-specific strategies

Not all individuals in a population may be equally capable of colour adjustments, with habitat and resource use differing across morphs. In Pacific tree frogs (*Hyla regilla*), individuals occur in either fixed green or brown morphs, or a separate morph capable of relatively slow colour change [52]. These morphs seem to represent different camouflage strategies for dealing with seasonal changes in a heterogeneous habitat.

Other species also show segregation into plastic and fixed morphs associated with spatial occurrence and habitat use, and potentially camouflage strategy. In the algal-dwelling shrimp *Hippolyte obliquimanus*, individuals occur in either a homogeneous morph (H), capable of colour change within a few days, or a striped translucent morph (ST), in which colour pattern seems relatively fixed [28]. In nature, H individuals occur in high densities against certain weed species, whereas ST individuals are more evenly distributed among various habitats [53]. Habitat fidelity is higher and swimming activity lower in H shrimp compared with ST individuals, and, correspondingly, ST shrimp have a more pelagic streamlined shape. These morphs may reflect different camouflage strategies, with H individuals well concealed to specific background types at any given time, and ST individuals relying more on transparency to cope with their more mobile lifestyle [28].

Similar morph-specific differences occur in other species. Many grasshoppers, for example, occur in fire-affected habitats, whereby melanic morphs camouflaged against burnt ground and lighter morphs against fresh vegetation arise through developmental plasticity based on background brightness [24]. Coupled with dispersal, this can allow for movement into new patches. In some pygmy grasshoppers (*Tetrix*), there is also evidence for differences in dispersal ability and dietary niches between morphs [54]. Such linked polymorphic states of coloration and behaviour may allow individuals to exploit different habitat types and reduce competition.

## 4. Concluding remarks and key future research

Below we outline 10 interconnected areas of camouflage and colour change that we feel are most important for future work to address.

(i) *When do visual processes underlie colour change for camouflage and what are the specific pathways?* Clearly, for many species, visual feedback is key to guiding colour change. Yet some animals also appear to rely on dietary factors. Generally, visual feedback should be important when colour change occurs in the short term and when future background environments are unpredictable. For example, cuttlefish moving rapidly to new patches should require visual information to change appropriately, as should juvenile crabs that might settle in a wide range of habitats. By contrast, species that consume what they live on and change via diet (e.g. some caterpillars), or where changes in background colour are predictable (e.g. snow cover and hares) or indicated by other cues (e.g. odour) may not need visual feedback.
(ii) *What are the physiological costs of colour change?* It is widely assumed that colour change carries energetic or metabolic costs. We would predict that costs would be dependent on the speed and type of change. Animals that change rapidly and continuously likely incur some costs, whereas in species that change slowly or with a dietary component, the costs may be small. Quantification of metabolic rate over different timescales for individuals induced to change colour versus those that are not are needed.
(iii) *What are the adaptive functions of circadian rhythms of colour change?* Rhythms are common in crustaceans, amphibians, reptiles and beyond, yet their functional significance (and mechanisms) are poorly understood. In some species, rhythms may facilitate thermoregulation and UV protection, or simply energy saving, whereas in others, rhythms allow concealment over 24 h.
(iv) *How do behavioural adaptations complement (or conflict with) colour change for camouflage and how are these behaviours controlled?* The significance of behavioural choice is striking for animals that change colour because, in many cases, substrate preferences should no longer be fixed, but like appearance be flexible. We would expect animals with very rapid and effective colour change to lack strong behavioural preferences (e.g. cephalopods), whereas in slow changing species and those living in situations where the habitat patches are large compared with body size (e.g. caterpillars, grasshoppers, crustaceans), substrate preferences linked to coloration should exist. Experiments should compare if and how behavioural preferences change when individuals are induced to match different substrates.
(v) *To what extent does colour change improve camouflage and increase survival chances?* Visual modelling or experiments using artificial prey remain the most common ways to test the value of camouflage. However, direct measurements of the survival benefits achieved by colour change, including risk during transitional phases, using real predators and prey in the field or laboratory are much needed.
(vi) *What camouflage strategies are used by colour-changing animals?* Studies subjectively describing the occurrence of distinct camouflage types, such as background matching or disruptive coloration, are common for species with fixed coloration. However, much less is known regarding the use and value of different strategies in colour-changing species. Experiments testing the response of animals when presented with different visual scenes, coupled with measurements of background matching, disruptive coloration and masquerade, would reveal when different types of camouflage are used. In general, species that have a high degree of plasticity and the potential for highly patterned appearance make ideal systems for testing this issue.
(vii) *Can colour change allow animals to resolve conflicting selection pressures?* Many species are frequently exposed to multiple predators, differing in their sensory systems and modes of attack, and change with various ecological parameters and age. Colour change may be a valuable solution to deal with multiple pressures. Future work should explore the life-history factors that predict changes from one defensive strategy to another. Insect larvae may be particularly useful systems, given that different instars frequently change between types of defence.
(viii) *How important are phylogenetic constraints in shaping patterns of colour change abilities across taxa?* Many species across multiple phyla change colour over varied timescales. Controlled phylogenetic comparative analyses are greatly needed to identify the driving forces and constraints on colour change evolution and the patterns observed. Potentially valuable groups to explore these issues are crustaceans and fish because they show diversity in colour change abilities among species, and because colour change occurs over varied temporal scales that may be linked to differing life-history factors.
(ix) *What factors underlie ontogenetic changes in appearance?* In many species, individuals vary in appearance through ontogeny. Understanding this could help us comprehend the driving factors behind colour change, ranging from species life history and ecology through to predator avoidance behaviour. Crustaceans and insect larvae are good candidates for such work, given the common ontogenetic changes that they display.
(x) *How common are discrete morphs in colour-changing species and why do they exist?* It is often assumed that all individuals of a colour-changing species have this ability, yet recent work shows the presence of alternative fixed morphs in some species. How widespread within-species alternative morphs are, how they differ in strategy and habitat use, and even in reproductive behaviour, are issues of widespread importance. In some cases, morphs may represent alternative ways of exploiting varying resources, with knock-on effects for population densities, competition and migration among habitats and patches.

Colour change involves multiple, often interconnected, mechanisms, including processes and pathways that we are yet to properly understand. In many species, a suite of mechanisms and selection pressures will be driving the ultimate appearance and adaptive value of colour forms. Despite substantial progress, there is much left to understand and this subject has much to reveal about key questions in ecology, physiology and evolution.

## References

[1] Poulton EB 1890 The colours of animals: their meaning and use. Especially considered in the case of insects, 2nd edn London, UK: Kegan Paul, Trench Trübner, & Co. Ltd.

[2] Wallace AR 1867 Mimicry and other protective resemblances among animals. Westminster Rev. 1 July, 1–43.

[3] Poulton EB 1903 Experiments in 1893, 1894, and 1896 upon the colour-relation between lepidopterous larvae and their surroundings, and especially the effect of lichen-covered bark upon *Odontopera bidentata*, *Gastropacha quercifolia*, etc. Trans. Entomol. Soc. Lond. 1903, 311–374. (10.1111/j.1365-2311.1903.tb01141.x)

[4] Stuart-Fox D, Moussalli A 2009 Camouflage, communication and thermoregulation: lessons from colour changing organisms. Phil. Trans. R. Soc. B 364, 463–470. (10.1098/rstb.2008.0254) 19000973 PMC2674084

[5] Caro T, Sherratt TN, Stevens M 2016 The ecology of multiple colour defences. Evol. Ecol. 30, 797–809. (10.1007/s10682-016-9854-3)

[6] Stevens M. 2016 Color change, phenotypic plasticity, and camouflage. Front. Ecol. Evol. 4, 51 (10.3389/fevo.2016.00051)

[7] Keeble FW, Gamble FW 1899 The colour-physiology of *Hippolyte varians*. Proc. R. Soc. Lond. B 65, 461–468. (10.1098/rspl.1899.0059)

[8] Bagnara JT, Hadley ME 1973 Chromatophores and color change. Englewood Cliffs, NJ: Prentice-Hall.

[9] Bagnara JT, Matsumoto J 2006 Comparative anatomy and physiology of pigment cells in nonmammalian tissues. In The pigmentary system: physiology and pathophysiology, 2nd edn (eds Nordlund JJ, Boissy RE, Hearing VJ, King RA, Oetting WS, Ortonne J-P). Oxford, UK: Blackwell.

[10] Ligon RA, McCartney KL 2016 Biochemical regulation of pigment motility in vertebrate chromatophores: a review of physiological color change mechanisms. Curr. Zool. 62, 237–252. 29491911 10.1093/cz/zow051 PMC5804272

[11] Umbers KDL, Fabricant SA, Gawryszewski FM, Seago AE, Herberstein ME 2014 Reversible colour change in Arthropoda. Biol. Rev. 89, 820–848. (10.1111/brv.12079) 24495279

[12] Noor MAF, Parnell RS, Grant BS 2008 A reversible color polyphenism in American peppered moth (*Biston betularia cognataria*) caterpillars. PLoS ONE 3, e3142 (10.1371/journal.pone.0003142) 18769543 PMC2518955

[13] Chiao CC, Wickiser JK, Allen JJ, Genter B, Hanlon RT 2011 Hyperspectral imaging of cuttlefish camouflage indicates good color match in the eyes of fish predators. Proc. Natl Acad. Sci. USA 108, 9148–9153. (10.1073/pnas.1019090108) 21576487 PMC3107294

[14] Kang C, Kim YE, Jang Y 2016 Colour and pattern change against visually heterogeneous backgrounds in the tree frog *Hyla japonica*. Sci. Rep. 6, 22601 (10.1038/srep22601) 26932675 PMC4773871

[15] Stevens M, Lown AE, Denton AM 2014 Rockpool gobies change colour for camouflage. PLoS ONE 9, e110325 (10.1371/journal.pone.0110325) 25333382 PMC4198244

[16] Stevens M, Lown AE, Wood LE 2014 Colour change and camouflage in juvenile shore crabs *Carcinus maenas*. Front. Ecol. Evol. 2, 14 (10.3389/fevo.2014.00014)

[17] Stuart-Fox D, Moussalli A, Whiting MJ 2008 Predator-specific camouflage in chameleons. Biol. Lett. 4, 326–329. (10.1098/rsbl.2008.0173) 18492645 PMC2610148

[18] Fingerman M 1973 Behavior of chromatophores of the fiddler crab *Uca pugilator* and the dwarf crayfish *Cambarellus shufeldti* in response to synthetic *Pandalus* red pigment-concentrating hormone. Gen. Comp. Endocr. 20, 589–592. (10.1016/0016-6480(73)90093-2) 4715241

[19] Powell BL 1962 The responses of the chromatophores of *Carcinus maenas* (L. 1758) to light and temperature. Crustaceana 4, 93–102. (10.1163/156854062X00120)

[20] Stevens M, Pei Rong C, Todd PA 2013 Colour change and camouflage in the horned ghost crab *Ocypode ceratophthalmus*. Biol. J. Linn. Soc. 109, 257–270. (10.1111/bij.12039)

[21] Rodgers GM, Gladman NW, Corless HF, Morrell LJ 2013 Costs of colour change in fish: food intake and behavioural decisions. J. Exp. Biol. 216, 2760–2767. (10.1242/jeb.080879) 23619415

[22] Hanlon RT, Chiao C-C, Mäthger LM, Barbosa A, Buresch KC, Chubb C 2009 Cephalopod dynamic camouflage: bridging the continuum between background matching and disruptive coloration. Phil. Trans. R. Soc. B 364, 429–437. (10.1098/rstb.2008.0270) 19008200 PMC2674088

[23] Kelman E, Tiptus P, Osorio D 2006 Juvenile plaice (*Pleuronectes platessa*) produce camouflage by flexibly combining two separate patterns. J. Exp. Biol. 209, 3288–3292. (10.1242/jeb.02380) 16916964

[24] Burtt E 1951 The ability of adult grasshoppers to change colour on burnt ground. Proc. R. Entomol. Soc. Lond. 26, 45–49. (10.1111/j.1365-3032.1951.tb00119.x)

[25] Fingerman M, Yamamoto Y 1967 Daily rhythm of melanophoric pigment migration in eyestalkless fiddler crabs, *Uca pugilator* (Bosc). Crustaceana 12, 303–319. (10.1163/156854067X00279)

[26] Shibley GA 1968 Eyestalk function in chromatophore control in a crab, *Cancer magister*. Physiol. Zool. 41, 268–279. (10.1086/physzool.41.3.30155460)

[27] Gillespie RG 1989 Diet-induced color change in the Hawaiian happy-face spider *Theridion grallator* (Araneae, Theridiidae). J. Arachnol. 17, 171–177. 10.1111/j.1558-5646.2012.01653.x 22946805

[28] Duarte RC, Stevens M, Flores AAV 2016 Shape, colour plasticity, and habitat use indicate morph-specific camouflage strategies in a marine shrimp. BMC Evol. Biol. 16, 218 (10.1186/s12862-016-0796-8) 27756220 PMC5070350

[29] Greene E 1996 Effect of light quality and larval diet on morph induction in the polymorphic caterpillar *Nemoria arizonaria* (Lepidoptera: Geometridae). Biol. J. Linn. Soc. 58, 277–285. (10.1111/j.1095-8312.1996.tb01435.x)

[30] Darnell MZ 2012 Ecological physiology of the circadian pigmentation rhythm in the fiddler crab *Uca panacea*. J. Exp. Mar. Biol. Ecol. 426–427, 39–47. (10.1016/j.jembe.2012.05.014)

[31] Gamble FW, Keeble FW 1900 *Hippolyte varians*: a study in colour-change. J. Cell Sci. 43, 589–698.

[32] Kleinholz LH, Welsh JH 1937 Colour changes in *Hippolyte varians*. Nature 140, 851–852. (10.1038/140851b0)

[33] Lovell PG, Ruxton GD, Langridge KV, Spencer KA 2013 Individual quail select egg-laying substrate providing optimal camouflage for their egg phenotype. Curr. Biol. 23, 260–264. (10.1016/j.cub.2012.12.031) 23333313

[34] Hultgren KM, Stachowicz JJ 2010 Size-related habitat shifts facilitated by positive preference induction in a marine kelp crab. Behav. Ecol. 21, 329–336. (10.1093/beheco/arp192)

[35] Ryer CH, Lemke JL, Boersma K, Levas S 2008 Adaptive coloration, behavior and predation vulnerability in three juvenile north Pacific flatfishes. J. Exp. Mar. Biol. Ecol. 359, 62–66. (10.1016/j.jembe.2008.02.017)

[36] Chassard C 1956 Polymorphisme des populations d’*Hippolyte varians* Leach et comportement en function de leur adaptation chromatique presente. B. Soc. Zool. Fr. 81, 413–418.

[37] Gilby BL, Mari RA, Bell EG, Crawford EW, Jun D, Lederer BI, Tibbetts IR, Burfeind DD 2015 Colour change in a filefish (*Monacanthus chinensis*) faced with the challenge of changing backgrounds. Environ. Biol. Fish. 98, 2021–2029. (10.1007/s10641-015-0424-2)

[38] Vignieri SN, Larson JG, Hoekstra HE 2010 The selective advantage of crypsis in mice. Evolution 64, 2153–2158. (10.1111/j.1558-5646.2010.00976.x) 20163447

[39] Kettlewell HBD 1955 Selection experiments on industrial melanism in the Lepidoptera. Heredity 9, 323–342. (10.1038/hdy.1955.36)

[40] Hultgren KM, Mittelstaedt H 2015 Color change in a marine isopod is adaptive in reducing predation. Curr. Zool. 61, 739–748. (10.1093/czoolo/61.4.739)

[41] Stevens M, Merilaita S 2011 Animal camouflage: from mechanisms to function. Cambridge, UK: Cambridge University Press.

[42] Stevens M, Cuthill IC 2006 Disruptive coloration, crypsis and edge detection in early visual processing. Proc. R. Soc. B 273, 2141–2147. (10.1098/rspb.2006.3556) PMC1635512 16901833

[43] Palma AT, Steneck RT 2001 Does variable coloration in juvenile marine crabs reduce risk of visual predation? Ecology 82, 2961–2967. (10.1890/0012-9658%282001%29082%5B2961%3ADVCIJM%5D2.0.CO%3B2)

[44] Stevens M, Wood LE, Lown AE 2014 Camouflage and individual variation in shore crabs (*Carcinus maenas*) from different habitats. PLoS ONE 9, e115586 (10.1371/journal.pone.0115586) 25551233 PMC4281232

[45] Todd PA, Qiu W, Chong KY 2009 Ontogenetic shifts in carapace patterning and/or colouration in intertidal and subtidal brachyuran crabs. Raffles B. Zool. 57, 543–550.

[46] Langridge KV, Broom M, Osorio D 2007 Selective signalling by cuttlefish to predators. Curr. Biol. 17, 1044–1045. (10.1016/j.cub.2007.10.028) 18088584

[47] Booth C 1990 Evolutionary significance of ontogenetic colour change in animals. Biol. J. Linn. Soc. 40, 125–163. (10.1111/j.1095-8312.1990.tb01973.x)

[48] Anderson JR, Spadaro AJ, Baeza JA, Behringer DC 2013 Ontogenetic shifts in resource allocation: colour change and allometric growth of defensive and reproductive structures in the Caribbean spiny lobster *Panulirus argus*. Biol. J. Linn. Soc. 108, 87–98. (10.1111/j.1095-8312.2012.01998.x)

[49] Wilson D, Heinsohn R, Legge S 2006 Age- and sex-related differences in the spatial ecology of a dichromatic tropical python (*Morelia viridis*). Austral. Ecol. 31, 577–587. (10.1111/j.1442-9993.2006.01519.x)

[50] Karlsson M, Johansson J, Caesar S, Forsman A 2009 No evidence for developmental plasticity of color patterns in response to rearing substrate in pygmy grasshoppers. Can. J. Zool. 87, 1044–1051. (10.1139/Z09-097)

[51] Bond AB, Kamil AC 2006 Spatial heterogeneity, predator cognition, and the evolution of color polymorphism in virtual prey. Proc. Natl Acad. Sci. USA 103, 3214–3219. (10.1073/pnas.0509963103) 16481615 PMC1413896

[52] Wente WH, Phillips JB 2003 Fixed green and brown color morphs and a novel color-changing morph of the Pacific tree frog *Hyla regilla*. Am. Nat. 162, 461–473. (10.1086/378253) 14582008

[53] Duarte RC, Flores AAV 2017 Morph-specific habitat and sex distribution in the caridean shrimp *Hippolyte obliquimanus*. J. Mar. Biol. Assoc. UK 97, 235–242. (10.1017/S0025315416000230)

[54] Karpestam E, Forsman A 2013 Stable isotopes reveal dietary divergence between dispersal phenotypes in *Tetrix subulata* pygmy grasshoppers (Orthoptera: Tetrigidae). Eur. J. Entomol. 110, 65–70. (10.14411/eje.2013.008)

